# Novel bilayer 2D V_2_O_5_ as a potential catalyst for fast photodegradation of organic dyes

**DOI:** 10.1038/s41598-024-65421-6

**Published:** 2024-06-24

**Authors:** P. R. Reshma, Arun K. Prasad, Sandip Dhara

**Affiliations:** Materials Science Group, Indira Gandhi Centre for Atomic Research, A CI of Homi Bhabha National Institute, Kalpakkam, 603102 Tamil Nadu India

**Keywords:** Materials science, Materials for energy and catalysis

## Abstract

Two-dimensional (2D) materials have recently drawn interest in various applications due to their superior electronic properties, high specific surface area, and surface activity. However, studies on the catalytic properties of the 2D counterpart of V_2_O_5_ are scarce. In the present study, the catalytic properties of 2D V_2_O_5_ vis-à-vis bulk V_2_O_5_ for the degradation of methylene blue dye are discussed for the first time. The 2D V_2_O_5_ catalyst was synthesized using a modified chemical exfoliation technique. A massive increase in the electrochemically active surface area of 2D V_2_O_5_ by one order of magnitude greater than that of bulk V_2_O_5_ was observed in this study. Simultaneously, ~ 7 times increase in the optical absorption coefficient of 2D V_2_O_5_ significantly increases the number of photogenerated electrons involved in the catalytic performance. In addition, the surface activity of the 2D V_2_O_5_ catalyst is enhanced by generating surface oxygen vacancy defects. In the current study, we have achieved ~ 99% degradation of 16 ppm dye using the 2D V_2_O_5_ nanosheet catalysts under UV light exposure with a remarkable degradation rate constant of 2.31 min^−1^, which is an increase of the order of 10^2^ from previous studies using V_2_O_5_ nanostructures and nanocomposites as catalysts. Since the enhanced photocatalytic activity emerged from the surface and optical properties of the catalyst, the current study shows great promise for the future application of 2D V_2_O_5_ in photo- and electrocatalysis.

## Introduction

Two-dimensional (2D) materials exhibit exotic physical, electrical, and optical properties that vary from their bulk counterparts. By altering the number of layers, the properties of 2D materials can be tailored. This distinctiveness provides opportunities for controlling the surface, optical, magnetic, and electrical properties of 2D materials for a range of applications. 2D materials are excellent choices for applications involving surface redox reactions, such as catalysis and gas sensing, because of their very high specific surface area and density of surface active states^1–8^. For instance, graphene exhibits a high specific surface area of ~ 2600 m^2^/g, whereas it is ~ 8 m^2^/g for natural graphite^9^. In addition to the high surface-to-volume ratio^9^, the presence of edge defects in 2D structures, which are responsible for stabilizing the structure, enhances the catalytic properties. For example, graphene with zig-zag edges shows good catalytic activity^10^. Theoretically, edge defect states strongly increase the electronic density of states at the edges compared to the plane of 2D materials^11^. In addition, the presence of dislocations, vacancy defects, impurities, and functional groups can significantly increase the density of surface active states and, consequently, the catalytic activity of the materials^12^. The impressive mechanical stability, generally shown by 2D materials, offers high stability and durability as catalysts and catalyst supports. Additionally, materials such as graphene, which has high electrical conductivity, have been applied in electrocatalysis.

The effluent from the textile industry contains organic dye molecules that are potentially harmful to human health and the ecosystem. Textile dyes are non-biodegradable and generally highly stable under light, heat, and oxidizer exposure^13^. Generally, catalyst-mediated redox reactions are cost-effective methods for the degradation of textile dyes^14–16^. Semiconducting metal oxides with bandgaps in the range of visible and UV light energies, such as V_2_O_5_^17^, SnO_2_^18^, ZnO^19^, ZrO_2_^20^, and TiO_2_^21,22^, are generally used as photocatalysts. As a transition metal, V possesses multiple oxidation states^23^, and the vanadium oxide surface can undergo reversible redox reactions^24,25^. This property makes V_2_O_5_ a promising catalyst. In addition, V_2_O_5_ nanostructures with various morphologies and heterostructures of V_2_O_5_/rGO, and V_2_O_5_/graphene also show good catalytic performance^26–28^. Under ambient conditions, V_2_O_5_ crystalizes in its orthorhombic polymorph *α-*V_2_O_5_, a layered van der Waals crystal^29^. The surface of the V_2_O_5_ catalyst consists of three differently coordinated oxygen atoms: vanadyl (O_I_), bridge (O_II_), and chain (O_III_) oxygen (the number in the subscript indicative of the coordination number of the oxygen atom)^29^. The bridging oxygen connects the double V–O chains in the crystal structure along the *b*-axis. The *VO*_*4*_ sites on the *ab*-plane V_2_O_5_ surface are the active surface sites for catalysis^30^. The supported vanadium oxide catalysts on oxide surfaces also tend to show improved catalytic performance by controlling the specific activity of *VO*_*4*_ sites^30^.

Using 2D V_2_O_5_, one can significantly reduce the defect formation energy and increase the active surface area for the catalytic reaction, consequently improving the catalytic performance. However, the catalytic properties of the 2D counterpart of V_2_O_5_ have not been reported before. The present study thoroughly examined the catalytic property of 2D V_2_O_5_ for the degradation of organic dyes. The chemically exfoliated V_2_O_5_ nanosheets were used as catalysts in the current study. The photocatalytic performance of 2D V_2_O_5_ for the degradation of methylene blue (MB) dye was investigated and compared with that of bulk V_2_O_5_.

## Methods

### Synthesis and characterization of 2D V_2_O_5_ nanosheets

2D V_2_O_5_ nanosheets were synthesized by a modified chemical exfoliation method by altering the concentration of bulk V_2_O_5_ in formamide^29^. Previous reports have shown that V_2_O_5_ intercalated with formamide molecules can provide a stable suspension of tiny flakes of V_2_O_5_ in formamide medium^29,31^. For the synthesis, bulk V_2_O_5_ powder (Merck, 99.99%) was dispersed in formamide (Merck, 99%) for 24 h. To separate the exfoliated nanosheets, the dispersion of formamide-intercalated V_2_O_5_ was subjected to a 1 h ultrasonic treatment at room temperature. The resultant suspension was then coated on a quartz substrate by drop casting (for the photocatalysis study), followed by heating at 100 °C to completely remove the formamide molecules. Furthermore, the exfoliated nanosheets were annealed for three hours at 250 °C under a continuous flow of O_2_^29^. The substrate was replaced with SiO_2_/Si and high pure gold for AFM and Raman spectroscopic studies, respectively.

A multimode scanning probe microscope (INTEGRA, NT-MDT, Russia) was used to record the atomic force microscopy (AFM) topography and estimate the thickness of the exfoliated nanosheets coated on the SiO_2_/Si substrate. Intermittent contact mode was used for imaging. Atomic-resolution STEM images were collected using probe aberration-corrected Thermo Fisher Scientific Themis Z ultrahigh-resolution TEM at an acceleration voltage of 300 kV. Oxygen vacancy defects in the sample were identified using X-ray photoelectron spectroscopy (XPS) (SPECS Surface Nano Analysis GmbH, Germany). A monochromatic Al *K*_*α*_ X-ray source (1486.7 eV) was used for the XPS measurements. Ultraviolet‒visible (UV‒Vis) absorption spectroscopy (Avantes) was carried out in absorption mode to determine the band gap. UV‒Vis absorption spectra of the bulk and 2D V_2_O_5_ nanosheets were collected using a cuvette with a width of 1 cm and the same light source. Raman spectroscopy (InVia, Renishaw, UK) was carried out utilizing a 532 nm Nd:YAG solid-state laser as the excitation source in the backscattering geometry, an 1800 g/mm grating, and a thermoelectrically cooled charged coupled device as the detector.

### Estimation of specific surface area

The electrochemically active surface area (ECSA) of bulk and 2D V_2_O_5_ were calculated and compared. The ECSA was estimated from the non-Faradic capacitive current associated with double layer charging using the scan rate dependence of cycle voltammograms (CV)^32,33^. The experimental setup consists of a three-electrode system with a Pt counter electrode and Ag/AgCl as the reference electrode. In the present study, for bulk V_2_O_5_, a 5 mM dispersion of bulk V_2_O_5_ in propanol was taken. Around 630 µL was dispersed on carbon paper with dimensions of 1.5 cm × 1.5 cm. The volume of the sample dispersion was chosen to ensure a conformal coating on the carbon paper. For 2D V_2_O_5_, 250 µL of a 1 mM dispersion of exfoliated V_2_O_5_ nanosheets (45 µg of sample) in formamide was coated on a similar substrate. The CV of the samples with 1 M H_2_SO_4_ electrolyte was recorded (using PGSTAT 302 N, Metrohm Autolab e.v.), and the peak current, I_c,_ was taken from the non-Faradic region.

### Photocatalytic study

The degradation of MB (C_16_H_18_ClN_3_S (Merck India)) dye was examined to understand the photocatalytic properties of the bulk material and 2D V_2_O_5_. The V_2_O_5_ catalyst mixed with the dye was subjected to UV-C (254 nm, 9 W, PL-S, PHILIPS, Poland). The intensity of the light source was measured using a Lutron UV light meter (Taiwan) at a distance of 3 cm from the light source, and the intensity was 2.21 mWcm^2^. An aqueous solution of dye and bulk V_2_O_5_ (concentration 0.18 mg/mL) was prepared in the dark and maintained at adsorption–desorption equilibrium. For 2D V_2_O_5_, 5 mL of 2D V_2_O_5_ dispersion (around 0.9 mg) was deposited on a quartz substrate and dipped into dye solution^32^. To achieve adsorption–desorption equilibrium, the sample was kept in the dark. The amount of 2D V_2_O_5_ sample was selected so that, for 5 mL of stock solution, the catalyst amount maintained the same as that for the bulk V_2_O_5_ catalyst. Light source was then exposed upon the dye solution containing the catalyst for various durations. Five milliliters of the irradiated solution was then subjected to UV‒Vis spectrometry. By monitoring the MB dye absorption peak at 664 nm, the degradation of MB dye was studied. For the quantitative study, the UV‒Vis absorption spectra of the solutions were periodically recorded.

## Results and discussion

### Estimation of nanosheet thickness and atomic resolution STEM studies

**Figure 1:**
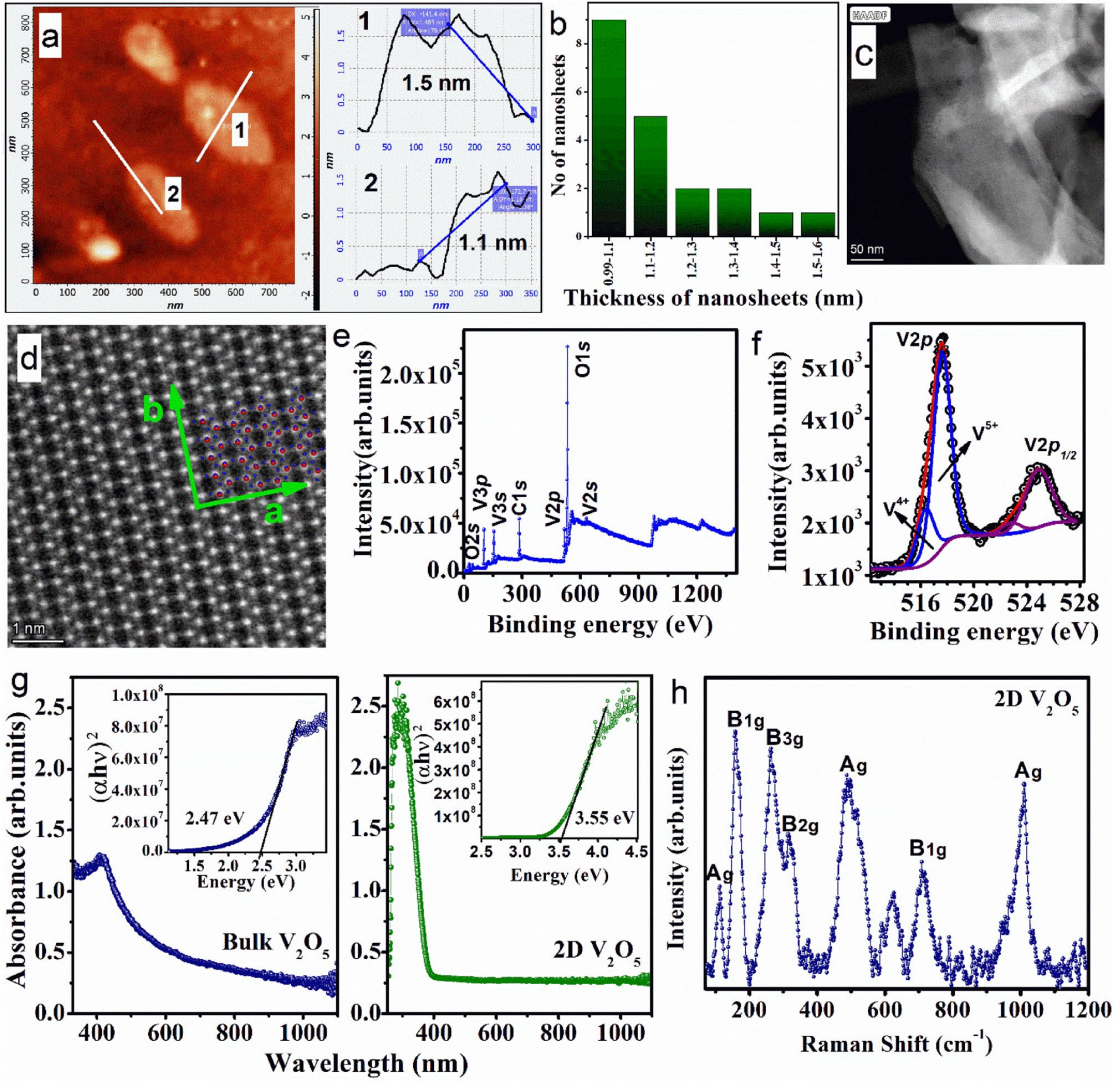
(**a**) AFM topography and height profile of exfoliated nanosheets. (**b**) Distribution of the thickness of 2D V_2_O_5_ nanosheets. (**c**) STEM image and (**d**) atomic resolution annular dark field (ADF)-STEM image of 2D V_2_O_5_ nanosheets. (**e**) XPS survey spectrum of 2D V_2_O_5_ and (**f**) the core-level spectrum corresponding to the binding energies of V*2p*_*3/2*_ for the V^5+^ and V^4+^ states of V atoms in 2D V_2_O_5_ nanosheets. (**g**) UV‒Vis absorption spectra of bulk and 2D V_2_O_5_. The Tauc plot for understanding the optical band gap is given in the corresponding insets. (**h**) Raman spectrum of the 2D V_2_O_5_ nanosheets.

AFM studies were carried out on the exfoliated V_2_O_5_ sample to determine the thickness. Figure 1a shows the AFM image of evenly distributed nanosheets on a Si/SiO_2_ substrate. The exfoliated layers in the ensemble have lateral dimensions between 100 and 300 nm, as observed from the FESEM image (Fig. S1). The observed step height has a standard deviation of 0.1 nm. The theoretical thicknesses of the monolayer, bilayer and trilayer V_2_O_5_ nanosheets are ~ 0.43, 0.87 and 1.3 nm, respectively^29^. The experimentally observed thicknesses of the individual layers of 2D V_2_O_5_ nanosheets are in the range of 1.0–1.5 nm, as shown in the height profile (Fig. 1a). The numerical distribution of the nanosheet thicknesses shown in Fig. 1b indicates that the quantity of bilayer thin nanosheets in the sample is substantially greater. To verify the atomic structure and chemical composition, an atomic resolution scanning transmission electron microscopy (STEM) image of the exfoliated nanosheets was obtained. Figure 1c and d show dark field (DF) STEM image and atomic resolution annular dark field (ADF)-STEM image of 2D nanosheets. The nanosheets in the DF-STEM image shown in Fig. 1c are folded, which is commonly observed in unsupported 2D structures^34^. The atomic resolution ADF image, displayed in Fig. 1d, is collected from the flat area of the individual nanosheets and demonstrates the arrangement of the VO_5_ square pyramidal structure in *α*-V_2_O_5_ along the (001) plane. The 2D layer is generated by joining the 1D chain of VO_5_ pyramids along the *b*-axis with chain oxygen (O_II_) along the *a*-axis.

### X-ray photoelectron spectroscopy studies

Figure 1e displays the XPS survey spectrum of 2D V_2_O_5_. The V_2_O_5_ phase of the exfoliated nanosheets was further validated by the observation of peaks at 517.4 (V*2p*_3/2_), 524.8 (V*2p*_1/2_), and 530.1 (O*1s*) in the XPS survey spectrum^35^. The deconvoluted peaks in the core-level spectrum observed at 517.4 and 516.3 eV corresponded to the binding energies of V*2p*_3/2_ for the V^5+^ and V^4+^ states, respectively (Fig. 1f). The peaks at 524.8 and 523.1 eV correspond to the binding energies of V*2p*_1/2_ for the V^5+^ and V^4+^ states, respectively^36^. The presence of the peaks corresponding to V^4+^ indicates the presence of oxygen vacancy sites in the samples^29^. The percentage of oxygen vacancy defects in 2D V_2_O_5_ was ~ 17%, as determined by the area under the curve. The presence of these oxygen vacancy sites can potentially increase the surface activity and, consequently, the catalytic property of the sample.

### UV‒Vis absorption studies for estimating the optical band gap

Bulk V_2_O_5_ is a semiconductor with a bandgap of ~ 2.4 eV and a Fermi energy between the O *2p* valence band and V *3d* conduction band^37^. Within this gap, however, two localized split-off bands exist approximately 0.6 eV below the main V *3d* band due to crystal field splitting^37^. The xy-derived levels of the conduction band are predicted to be at the lowest energies because they are not affected by vanadyl oxygen interactions. However, only two of the four xy-derived levels interact with the bridging oxygen and move up into the conduction band. Hence, the strong, indirect V-V interactions across the bridging oxygen are found to be the cause of the existence of split-off bands^37^. Figure 1g shows the UV‒Vis absorption spectra and Tauc plots for estimating the band gap for bulk and 2D V_2_O_5_. A significant increase in the absorbance value from 0.22 to 1.58 was seen between bulk and bilayer V_2_O_5_ (Fig. 1g). Additionally, there is a substantial increase in the optical absorbance for 2D and bulk V_2_O_5_ with the same concentration of sample and intensity of incident light. This observation is possibly due to the pronounced excitonic effects in bilayer 2D V_2_O_5_, which is also observed in other 2D materials arising out of the reduced dielectric screening effect^38,39^. This increase in the optical absorbance of 2D V_2_O_5_ indicates an exceptionally high number of photogenerated free charges, which can actively participate in the photocatalytic process. In addition, an absorption edge blueshift from bulk to bilayer 2D V_2_O_5_ was observed, indicating electronic decoupling with decreasing layer thickness in the 2D nanosheets of V_2_O_5_^29^. The optical bandgap and *E*_*g*_ of bulk and 2D V_2_O_5_ are experimentally calculated from Tauc’s Plot to be 2.47 and 3.55 eV, respectively (Fig. 1g). These results are in agreement with our earlier report on the synthesis of 2D nanosheets of V_2_O_5_^29^.

### Raman spectroscopic analysis of bilayer 2D V_2_O_5_

The Raman spectrum of the 2D V_2_O_5_ is given in Fig. 1h. The sample exhibited nine vibrational modes corresponding to 2D V_2_O_5_. The low-frequency vibrational modes A_1g_ and B_1g_ at 111 and 158 cm^−1^, respectively, are due to chain translation along the crystallographic *c-* and *a-*axes. V‒O_II_‒V bending vibration causes the B_3g_ mode at 262 cm^−1^. The deflection of V‒O_III_ and V‒O_III′_ along the *c* direction is responsible for the vibrational mode B_2g_ at 324 cm^−1^. The A_1g_ peak at 489 cm^−1^ arises from the bending vibration of V‒O_II_‒V in the *c* direction. The vibration of V‒O_III_′ and V‒O_III_ along the *a*-axis is the origin of the vibrational mode B_1g_ at 713 cm^−1^. The highest frequency A_1g_ mode at 1010 cm^−1^ corresponds to the stretching vibration of V‒O_I_ along the *c-*axis^29^. The peak at 620 cm^−1^ in the Raman spectrum is expected to arise due to surface oxygen vacancies and consequent surface reduction^29^.

### Electrochemical surface area of bulk and 2D V_2_O_5_

**Figure 2:**
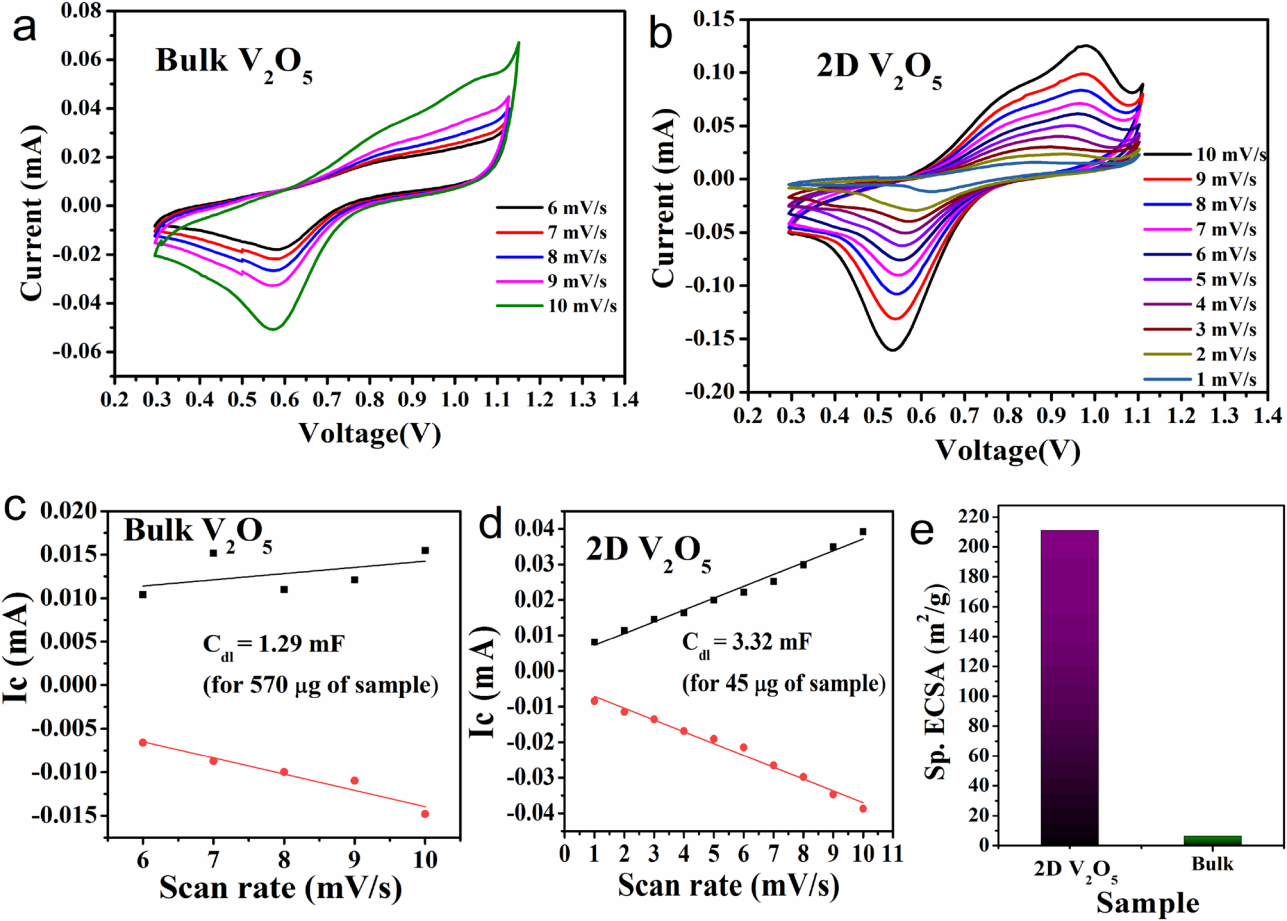
CV diagram of (**a**) bulk V_2_O_5_ and (**b**) 2D V_2_O_5_. Peak current versus scan rate in the non-Faradic region for (**c**) bulk V_2_O_5_ and (**d**) 2D V_2_O_5_. (**e**) ECSA for 1 g of bulk and 2D V_2_O_5_.

The specific surface area of the 2D V_2_O_5_ nanosheets was compared with that of bulk V_2_O_5_ by calculating the ECSA. The ECSA of a sample is essentially estimated from the electrochemical double-layer capacitance. The measurement can be performed either from the non-Faradic capacitive current associated with double-layer charging from the scan rate dependence of CV or from the frequency-dependent impedance of the system using electrochemical impedance spectroscopy^32,39^. In the present study, the first method was adopted for the measurement of the ECSA. The faradic process will be absent in the potential window of, typically, 0.1 V around the open circuit voltage. The CV curves of the samples were recorded using 1 M H_2_SO_4_ as the electrolyte. From the non-Faradic part, the peak current, Ic, is obtained. Thus, the ECSA may be determined using,

1 $$ECSA= \frac{{C}_{dl}}{{C}_{s}}$$

where *C*_*s*_ is the specific capacitance of an atomically smooth planar surface of the material per unit area under identical electrolyte conditions. For the 1 M H_2_SO_4_ electrolyte, the *C*_*s*_ were found to be 0.035 mF/cm^32,39,40^. Figure 2a and b show CV diagrams of bulk and 2D V_2_O_5_, respectively. The values of the double layer capacitance (C_dl_) are 1.29 mF for bulk, as shown in Fig. 2c, and 3.32 mF for 2D V_2_O_5,_ as shown in Fig. 2d. The corresponding ECSAs per 1 g of bulk and 2D V_2_O_5_ were determined to be 6.5 and 211 m^2^/g, respectively (Fig. 2e). According to the literature, bulk V_2_O_5_ has a shallow specific surface area < 10 m^2^/g^31,32^. Thus, the observed specific surface area of bulk V_2_O_5_ is in agreement with the literature. This study revealed that, in comparison with the bulk equivalent, 2D nanosheets of V_2_O_5_ exhibited an increase in specific surface area of approximately 32 times.

### Degradation of MB dye under UV irradiation

**Figure 3:**
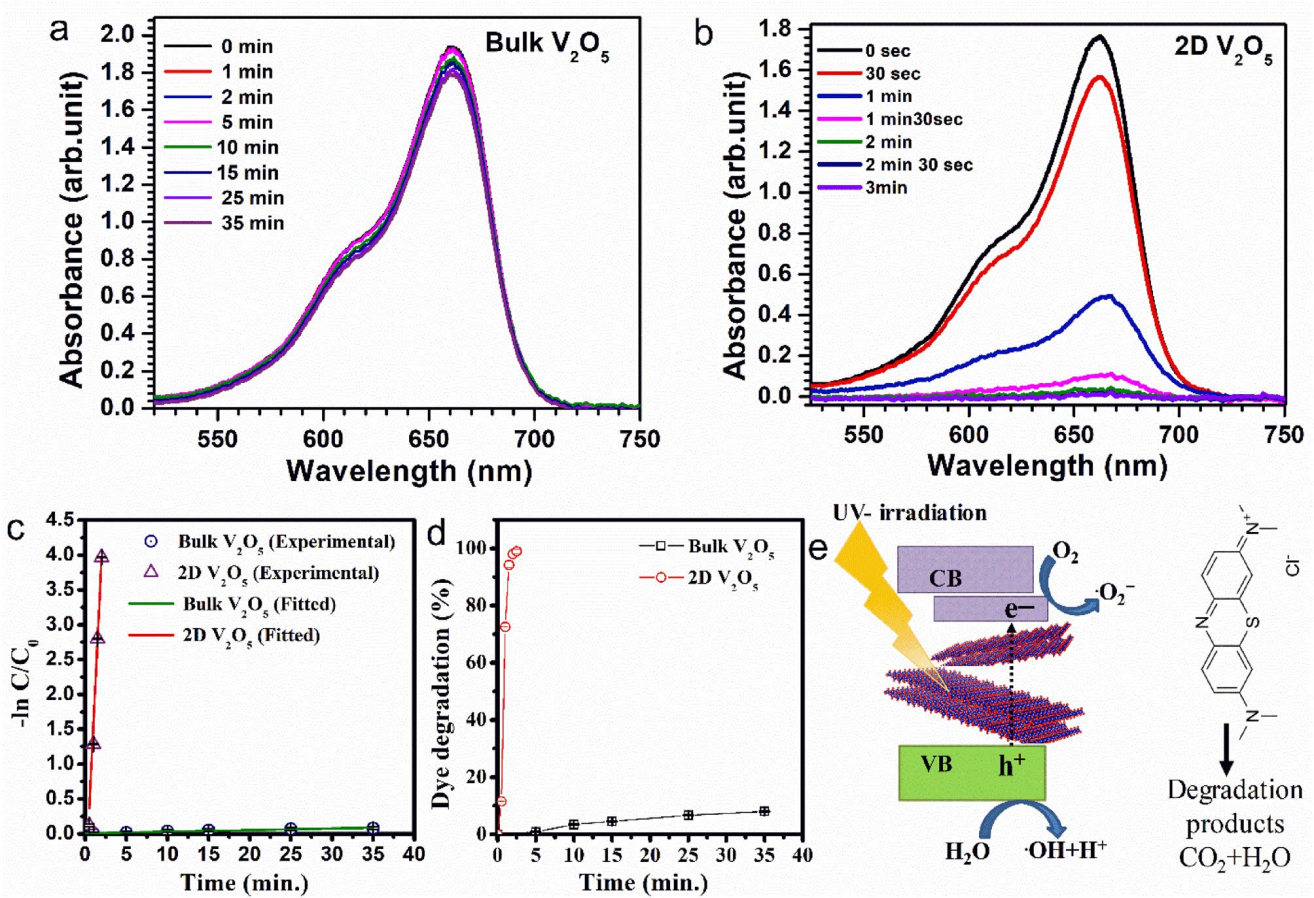
UV‒Vis absorption spectra of MB dye solutions for different durations with (**a**) bulk V_2_O_5_ and (**b**) 2D V_2_O_5_ as catalysts. (**c**) The curve of ln(C_0_/C_t_) vs. light exposure time (**d**) Dye degradation efficiency using the bulk and 2D V_2_O_5_ samples as catalysts versus the duration of light exposure. (**e**) Schematic of dye degradation mechanism of 2D V_2_O_5_.

The photodegradation of MB dye was studied using bulk and 2D V_2_O_5_ as catalysts. The UV‒Vis absorption spectra of aqueous dye solutions collected at various UV light exposure times containing the bulk and 2D V_2_O_5_ catalysts are displayed in Fig. 3a and b. The benzene ring and heteropoly aromatic bond of the MB dye molecule are broken upon exposure to UV light in the presence of the catalyst, which is apparent from the decrease in the strength of the absorption peak at 662 and 608 nm^32,41^. As a result, upon exposure to UV light in the presence of the catalyst, the dye molecules undergo degradation. The amount of catalyst was kept the same for all bulk and 2D V_2_O_5_ samples for the same amount of dye solution. The photodegradation of MB dye under UV light exposure without any catalysts was also tested and is shown in Fig. S2 (Supplementary information). The degradation of MB dye using 2D and bulk V_2_O_5_ catalysts was also studied under exposure to visible light, and the results are shown in the supplementary information (Fig. S3). Only ~ 5% of the dye underwent degradation with a UV light exposure of 160 min. As shown in Fig. 3a and b, the bulk V_2_O_5_ catalyst caused only minimal degradation of 7.7% after 35 min of UV exposure of the dye (16 ppm), whereas 2D V_2_O_5_ catalyst resulted in the degradation of ~ 99% in just 3 min.

### Estimation of the rate constant and efficiency of dye degradation

Generally, the degradation obeys pseudo-first-order kinetics^32,41,42^, and according to the observations, the degradation reaction fits well with the equation $\text{ln}\frac{{C}_{0}}{{C}_{t}}=kt$, where C_0_ is the absorption at t = 0, and C_t_ is the absorption after the time interval* t* of the irradiation (Fig. 3c). Based on Fig. 3c, the rate constants for dye degradation using the bulk and exfoliated V_2_O_5_ catalysts were calculated. The 2D and bulk V_2_O_5_ catalysts exhibit distinct rate constants for the degradation of MB dye: 2.311 min^−1^ and 0.0023 min^−1^, respectively.

The percentage (%) of photocatalytic dye degradation efficiency was calculated using Eq. (2)^43^.

2 $$D\left(\%\right)=\frac{{C}_{0}-{C}_{t}}{{C}_{0}} \times 100$$

Figure 3d depicts the effect of light exposure duration on degradation efficiency. As discussed in the previous sections, the specific surface areas (ECSA) of the bulk and 2D V_2_O_5_ are 6.5 and 211 m^2^/g, respectively. As a result of this increase in the specific surface area, the photocatalytic performance of the 2D nanosheets substantially improved compared with that of the bulk V_2_O_5_ catalyst. In addition, surface oxygen vacancies can act as active sites for redox reactions and enhance surface activity. A comparison of the reported literature with the present study (Table 1) also indicates the superior catalytic performance of 2D V_2_O_5_ nanosheets.

**Table 1:** Comparison of the present study on the photocatalytic degradation of MB dye using a 2D V_2_O_5_ catalyst with the reported literature on related materials as catalysts.

Photocatalyst	Rate constant min^−1^	Degradation time (min)	Efficiency (%)	Reference
V_2_O_5_ np	0.00742	20	31	^44^
rGO-V_2_O_5_	0.0184	20	71	^44^
rGO-V_2_O_5_	0.0078	255	85	^45^
V_2_O_5_-nanorods	0.00135	300	24	^46^
V_2_O_5_ thin film	–	210	75	^47^
V_2_O_5_ np	0.025	–	92	^48^
Graphene- V_2_O_5_	0.0367	90	98	^27^
Nd^3+^ doped V_2_O_5_	–	120	80	^49^
Ho-doped V_2_O_5_	–	160	93	^50^
Yb-doped V_2_O_5_	–	160	95	^50^
Na_2_Ti_3_O_7_/V_2_O_5_/*g*-C_3_N_4_	0.103	–	–	^51^
Na_2_Ti_3_O_7_/V_2_O_5_	0.0865	–	–	^52^
V_2_O_5_/rGO	0.048	100	98	^53^
2D V_2_O_5_	2.311	3	~ 99	Present study

### Effect of scavenging agents on dye degradation

**Figure 4:**
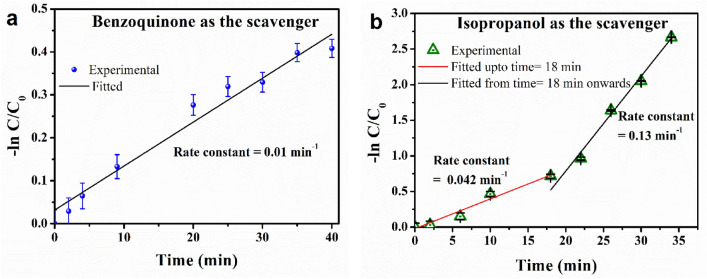
The curve of ln(C_0_/C_t_) vs. UV exposure time using 2D V_2_O_5_ as the catalyst in the presence of scavengers, (**a**) benzoquinone, and (**b**) isopropanol.

The MB dye contains active sites for oxidative attack. To understand the active oxidative species involved in the catalytic process, the effects of scavenging reagents in quenching superoxide (${\text{O}}_{2}^{-}$) and hydroxyl (^·^OH) radicals during the catalytic process were studied. *p-*Benzoquinone and isopropanol were used as the scavenging reagents for superoxide (${\text{O}}_{2}^{-}$) and hydroxyl (^·^OH) radicals, respectively. *p-*benzoquinone and isopropanol were added at concentrations of 10 mM and 20 mM, respectively. The catalytic degradation of the MB dye molecule was studied using the same method as described in the previous sections. Figure S3a and b show the UV‒Vis absorption spectra of MB dye molecules in the presence of the 2D V_2_O_5_ catalyst and the scavengers *p-*benzoquinone and isopropanol, respectively. Figure 4a and b show the rate constants obtained in the presence of *p-*benzoquinone and isopropanol, respectively. The rate constant of the catalytic degradation of MB dye decreased to 0.01 min^−1^ in the presence of *p-*benzoquinone, while it decreased to 2.31 min^−1^ without any scavengers. In the presence of isopropanol, the rate constant decreased to 0.04 min^−1^ during the first 18 min of UV light illumination, followed by a rate constant of 0.13 min^−1^. This change in the rate constant is due to the decrease in the isopropanol concentration due to evaporation. In the presence of both scavenging reagents, a significant reduction in the rate constant is observed. The change in rate constant indisputably proves that the ^.^${\text{O}}_{2}^{-}$ and ^·^OH radicals are involved in the photocatalytic reaction.

### Mechanism of photocatalytic degradation of MB dye

Figure 3e shows a schematic illustration of a possible pathway for the photocatalytic degradation of MB dye. When 2D V_2_O_5_ nanosheets are exposed to UV light, electrons are excited to the conduction band (CB), leaving holes in the valence band (VB). ^·^OH radicals are generated when these holes interact with OH^−^ on the catalyst surface. These electrons lead to the production of ${\text{O}}_{2}^{-}$ superoxide anion radicals. The effect of scavengers on dye degradation has demonstrated that the oxidizing species in the photocatalysis reaction are ^·^OH and ^**.**^${\text{O}}_{2}^{-}$ radicals that are thereby produced. According to the results, the use of 2D V_2_O_5_ nanosheets effectively improved the separation of electrons and holes, which substantially enhanced the photocatalytic activity. The anticipated decrease in the screening effect of charges and the presence of excitonic levels in 2D materials could also be responsible for this enhanced separation of electrons and holes^54^ in 2D V_2_O_5_.

### Recyclability of the catalyst

**Figure 5:**
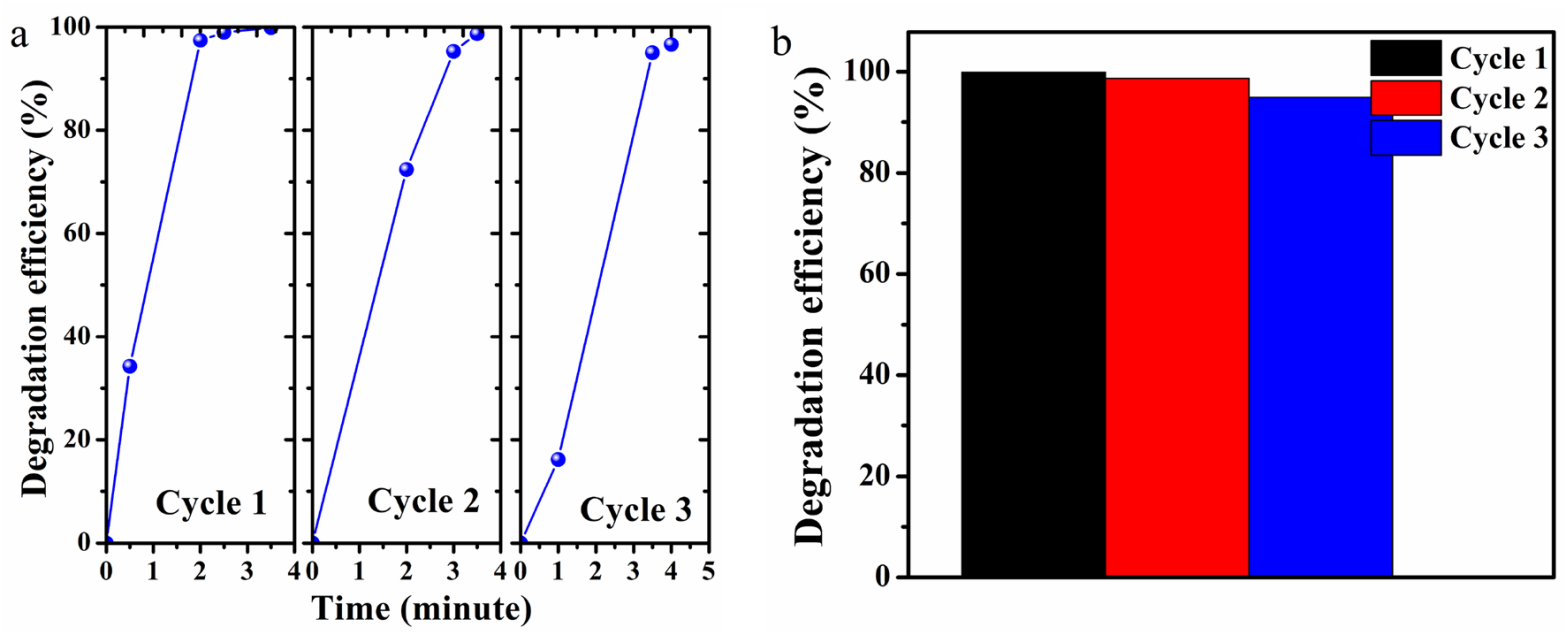
(**a**) MB dye degradation efficiency for three cycles of reuse of 2D V_2_O_5_ nanosheets. (**b**) Bar diagram showing the dye degradation efficiency after 3.5 min of UV light exposure for three cycles.

The recyclability of the 2D V_2_O_5_ catalyst was determined by reusing a 2D V_2_O_5_ sample coated on a quartz substrate. After each cycle, the sample on the substrate was collected, washed with deionized water (Millipore), and dried at 80 °C. Figure 5a shows the degradation efficiency of the catalyst under UV light illumination, and Fig. 5b shows the degradation efficiency of the 2D V_2_O_5_ catalyst under UV light illumination for 3.5 min over three cycles. The decrease in efficiency can be due to the loss of catalyst from the substrate during the recycling process.

## Conclusions

2D V_2_O_5_ nanosheets with a bilayer thickness were synthesized using a chemical exfoliation technique, which showed a ~ 30-fold increase in the specific surface area from that of the bulk. The catalytic activity of 2D V_2_O_5_ was studied in detail and compared with that of bulk V_2_O_5_. A considerable improvement in the degradation rate constant was observed for 2D V_2_O_5_ from bulk V_2_O_5_ catalysts. The rate constants for the bulk and 2D V_2_O_5_ catalysts were 0.0023 and 2.311 min^−1^, respectively. The sizable improvement in the catalytic activity is attributed to three properties:

- A massive increase in the specific surface area of 2D nanosheets from bulk V_2_O_5_ was observed as a result of the formation of nanosheets with thicknesses down to the bilayer.
- The presence of oxygen vacancies acts as an active site for redox reactions, improving the surface activity.
- An increase in the optical absorption coefficient on the order of ~ 7 times substantiates a significant increase in the number of photoexcited electrons. The efficient separation of photogenerated carriers facilitates the generation of free radicals responsible for the oxidative decomposition of organic dye molecules.

The results obtained from the present study undeniably prove that the novel 2D V_2_O_5_ nanosheet is a promising photocatalyst. In addition, the 2D V_2_O_5_ catalyst is stable and abundant on earth, the catalyst is recyclable, and the synthesis method of the catalyst is cost effective. Additionally, the current study opens up new possibilities for using 2D V_2_O_5_ as electro- and photocatalyst because of its improved surface and optical properties. The increase in optical absorption leads to the possibility of efficiently generating hot electrons, creating novel possibilities for light energy conversion to electrical and chemical energy.

### Supplementary Information

Supplementary Information.

## Data Availability

The datasets generated and analyzed during the current study are available from the corresponding author upon reasonable request.
