# Supplementary material for: Novel bilayer 2D V2O5 as a potential catalyst for fast photodegradation of organic dyes
Source: Sci Rep. 2024 Jun 24;14:14462. doi: 10.1038/s41598-024-65421-6 (PMC11196694; doi:10.1038/s41598-024-65421-6)
Supplement: Supplementary file 1 — Supplementary Information. [file 41598_2024_65421_MOESM1_ESM.docx]

Supplementary Information

Novel bilayer 2D V_2_O_5_ as a potential catalyst for fast photodegradation of organic dyes

*Reshma P R, Arun K Prasad, Sandip Dhara*

Materials Science Group, Indira Gandhi Centre for Atomic Research, A CI of Homi Bhabha National Institute, Kalpakkam 603102, Tamil Nadu, India

Corresponding authors: [reshmapattaniparambil@gmail.com](mailto:reshmapattaniparambil@gmail.com), [akp@igcar.gov.in](mailto:akp@igcar.gov.in)

**FESEM images**

The FESEM images of the bulk and 2D V_2_O_5_ samples are shown in Figure S1a and S1b, respectively. Figure S1a shows that the bulk V_2_O_5_ contains micron-sized particles. The average particle size estimated from the FESEM image of the bulk V_2_O_5_ particles is 1.6 μm. Figure S1b shows exfoliated V_2_O_5_ nanosheets with lateral dimensions in the range of 100-300 nm.





**Figure S1** FESEM images of a) bulk V_2_O_5_ and b) 2D V_2_O_5_. Scale bars are shown inside the respective images.

**Degradation of Methylene blue dye under UV exposure without a catalyst**

The photodegradation of MB dye under UV light exposure was tested without any catalysts. Only 5% dye degradation was observed with a UV light exposure of 160 min. This value is significantly slower than the degradation rate with the catalysts. Figure S2a shows the UV‒Vis absorption spectra of the aqueous dye solution without catalyst taken at particular time intervals of UV light exposure. Figure S2b shows the percentage of dye degradation with increasing UV light exposure time in the absence of any catalysts.





**Figure S2** a) UV‒Vis absorption spectra of methylene blue (MB) dye solutions with different UV light exposure times without any catalyst. b) Percentage of dye degradation with UV light exposure time without catalyst.





**Figure S3** UV‒Vis absorption spectra of methylene blue (MB) dye solutions with different light exposure times in the presence of a) benzoquinone as the scavenger, b) isopropanol as the scavenger and 2D V_2_O_5_ as the catalyst.

**Catalytic degradation of methylene blue dye under visible light exposure**

The photodegradation of MB dye under visible light exposure was also studied using bulk and 2D V_2_O_5_ as catalysts. Figures S3a and S3b show the UV‒Vis absorption spectra of aqueous dye solutions with bulk and 2D V_2_O_5_ catalysts taken at particular time intervals of visible light exposure. The V_2_O_5_ catalyst was used to degrade the dye under a light source (28.4 mW/cm^2^, 11 W, PL-S, PHILIPS, Poland) covering visible wavelengths from 400 to 750 nm. The intensity of the light source was measured using a Lutron UV light meter (Taiwan) at a distance of 3 cm from the light source, and the intensity was 2.21 mWcm^2^. An aqueous solution of dye and bulk V_2_O_5_ catalyst (0.9 mg of catalyst in 5 ml of stock solution) was made in the dark and maintained at adsorption-desorption equilibrium. For 2D V_2_O_5_, 0.9 mg of sample (5 ml of 1 mM sample dispersion in formamide) was coated on a quartz substrate, dipped into 5 ml of dye solution and kept in the dark for adsorption-desorption equilibrium.





**Figure S4** UV‒Vis absorption spectra of methylene blue (MB) dye solutions with different visible light exposure times in the presence of a) bulk V_2_O_5_ and b) 2D V_2_O_5_ as catalysts. c) The percentage of dye degradation with time for reaction in the presence of catalysts and without catalyst (blank). d) The curve of ln(C_0_/C_t_) vs. light exposure time for determining the rate constant of dye degradation in the presence of two catalysts and without the catalyst.

As shown in Figures S3a, S3b and S3c, the bulk V_2_O_5_ catalyst caused degradation of 8 ppm dye up to 66% with visible light exposure for 320 minutes. At the same time, the 2D V_2_O_5_ catalyst degraded ~ 81% of the 8 ppm dye upon exposure to visible light for 320 min. The rate constants of MB dye degradation by the bulk and 2D V_2_O_5_ catalysts were 0.0028 and 0.004 min^-1^, respectively (Figure S3d). Compared with that of the bulk V2O5 catalyst, the catalytic degradation under visible light exposure still showed a slightly greater degradation constant for 2D V_2_O_5_. The increased degradation rate of 2D V_2_O_5_ in visible light exposure is possible because of the presence of defects in the 2D V_2_O_5_ catalyst, which creates mid-band states within the visible light energy range. [1]





**Figure S5** The absorption spectra of methylene blue dye with the catalyst under dark conditions.

Figure S5 shows the UV‒Vis absorption spectra of the dye solution and the solution supplemented with the 2D V_2_O_5_ catalyst and kept under dark conditions for over 27 h under centrifugation. A decrease in the absorption peak signifies the adsorption of some of the dye molecules on the catalyst surface after the adsorption-desorption equilibrium is reached. Each photocatalyst measurement in the current study was performed after setting the dye-catalyst solution to adsorption-desorption equilibrium.

**References**

1. Juine, R.N., B.K. Sahu, and A. Das, *Recyclable ZnS QDs as an efficient photocatalyst for dye degradation under the UV and visible light.* New Journal of Chemistry, 2021. **45**(13): p. 5845-5854.
